# Clinical and angiographic characteristics of patients with familial hypercholesterolemia presenting with ST-elevation myocardial infarction undergoing primary percutaneous coronary intervention

**DOI:** 10.1038/s41598-024-77656-4

**Published:** 2024-11-07

**Authors:** Khaled M. Elmaghraby, Ahmed Abdel-Galeel, Amira Harby Osman, Hosam Hasan-Ali, Mohamed Aboel-Kassem F. Abdelmegid

**Affiliations:** Cardiovascular Medicine Department, Assiut University Heart Hospital, Assiut University, Assiut, 71526 Egypt

**Keywords:** Cardiology, Dyslipidaemias

## Abstract

Familial hypercholesterolemia (FH) is a world public health problem that enhances the risk of premature coronary artery disease (CAD) with a high incidence of acute coronary syndrome. This study aimed to evaluate the clinical and angiographic characteristics of the patients with and without FH who had ST-elevation myocardial infarction (STEMI). It included 690 patients who presented with the first attack of STEMI and underwent primary percutaneous coronary interventions (PPCI). The patients were analyzed to diagnose FH according to the Dutch Lipid Clinic Network (DLCN) criteria. All angiograms were analyzed for the number of diseased vessels, Syntax score, thrombus burden grade, and final Thrombolysis in Myocardial Infarction (TIMI) flow grade. The majority of patients were male (72.6%) with a mean age of 54 ± 12 years. Based on DLCN criteria, they were classified into unlikely/possible FH (86.1%) and probable/definite FH (13.9%) groups. Probable/definite FH patients were significantly younger, and higher incidence of males < 55 years compared with unlikely/possible FH patients (*p* < 0.001 for each). Moreover, probable/definite FH patients had a higher frequency of three-vessel disease (*p* = 0.007) and Syntax score (*p* < 0.001) with a moderate positive correlation with the DLCN score (*r* = 0.592, *p* < 0.001). Furthermore, probable/definite FH patients showed a higher thrombus burden and final TIMI slow/no-reflow when compared to the unlikely/possible FH patients (*p* = 0.006 and *p* = 0.027, respectively). Patients with probable/definite FH and LDL-C level were independent predictors of high thrombus burden besides males < 55 years, and the number of diseased vessels. In conclusion, STEMI patients with FH were younger males and associated with severe CAD with frequent multivessel CAD, high anatomical complexity of CAD, and frequent high thrombus burden. Furthermore, FH was one of the predictors of high thrombus burden.

## Introduction

Familial hypercholesterolemia (FH) is a common autosomal dominant genetic disease characterized by elevated low-density lipoprotein cholesterol (LDL-C) levels^1^. The most often used criteria for diagnosis of FH are the Dutch Lipid Clinic Network (DLCN) criteria^2^.

FH increases the risk of premature cardiovascular disease (CVD), mainly CAD, and premature death at a young age^3,4^. The incidence of acute coronary syndrome is 15–20 times higher in patients with untreated FH compared to those without FH^5,6^. In addition, FH patients were at a 2.34-fold increased risk of recurrent acute coronary syndrome than those without FH^7^. Therefore, LDL-C is important in the assessment of acute coronary syndrome patients^8^.

Many studies demonstrated that patients with FH had more severe CAD than patients without FH using the Gensini score^9–11^. Few studies showed that FH increases the complexity of CAD lesions^12^. However, in FH patients presenting with ST-elevation myocardial infarction (STEMI), there is limited data about the complexity of CAD, thrombus burden grade, and final Thrombolysis in Myocardial Infarction (TIMI) flow grade that affects the patient’s prognosis. Also, clinical data on FH patients with STEMI is still relatively scarce.

Therefore, this study’s objectives were; first, to compare the demographic and clinical features of patients with FH who have experienced STEMI to those without FH. Second, to evaluate the angiographic characteristics of these patients, focusing on the severity and anatomical complexity of coronary artery lesions and the grade of coronary thrombus burden.

## Methods

### Study population

The present study included patients who presented with the first attack of acute STEMI (type 1 myocardial infarction) at Assiut University Heart Hospital and underwent PPCI between August 2020 and July 2021. All patients were diagnosed with acute STEMI (type 1 myocardial infarction) according to the fourth universal definition of myocardial infarction^13^ and were eligible for PPCI. Patients with acute STEMI who were referred for coronary bypass graft surgery, receiving fibrinolytic therapy, undergoing conservative strategy, had prior use of antihyperlipidemic drugs, or had clinical and laboratory evidence of secondary hyperlipidemia (such as hypothyroidism, nephrotic syndrome, chronic kidney disease, Cushing’s syndrome, pheochromocytoma, or obstructive jaundice) were excluded from the study.

## Sample size calculation

Based on the previous estimated prevalence of the FH in patients presenting with myocardial infarction, an estimated minimum sample size was 342 patients which was multiplied by 2 design effect to compensate for not using a simple random sampling technique. Therefore, a net sample was 684 patients needed to carry out this study. The following equation for the sample size for descriptive study design^14^ was used:

$$\:Sample\:size\:=\:{\left({Z}_{1}-\alpha/2\right)}^{2}P(1-P)/{D}^{2}$$

*Z*_*1 – α /2*_ represents the number of standard errors from the mean for the corresponding level of confidence. (At 95% CI or 5% level of significance (type-I error) it is 1.96 and at 99% CI it is 2.58)

*P* is the expected prevalence based on previous research^6^.

*D* is the margin of error or precision; the absolute precision required on either side of the proportion.

## Study design

A detailed history, physical examination, 12-lead electrocardiogram (ECG), and blood sample were taken upon the patient’s arrival at the hospital. All patients underwent a PPCI by an expert interventional cardiologist. Only the infarct-related artery was targeted. All angiograms were reviewed and analyzed in the core laboratory by two interventional cardiologists blinded to all data. From the worst angiographic view, the lesions were evaluated visually in several projections. Significant CAD was defined as stenosis of ≥ 60% of one major coronary artery or ≥ 50% of the left main coronary artery. Patients were classified as having 1, 2, or 3 vessels by counting the number of affected vessels. Stenosis of ≥ 50% in the left main coronary artery was considered a 2-vessel disease.

The Syntax Score was used to assess the complexity of coronary artery disease^15^. It was calculated for each patient by scoring all the coronary artery lesions with diameter stenosis ≥ 50% in vessels ≥ 1.5 mm using a web-based (http://www.syntaxscore.org) or smartphone application.

**Fig. 1:**
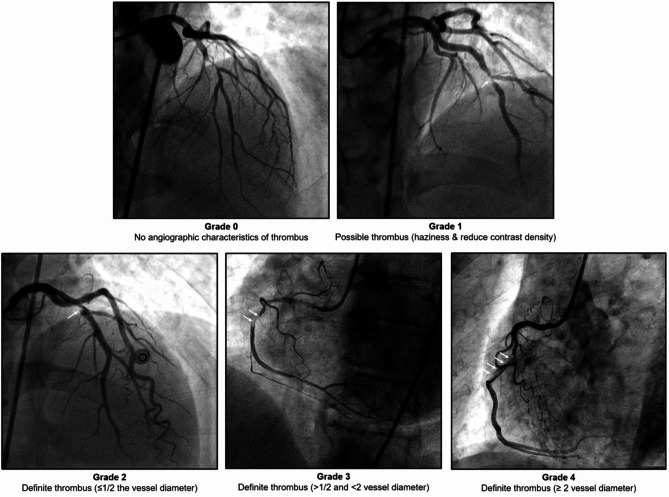
Intracoronary thrombus burden classification grades.

Intracoronary thrombus burden was angiographically assessed and classified into the following grades (Fig. 1); in grade 0, no angiographic characteristics of thrombus are present; in grade 1, a possible thrombus is present; in grade 2, there is a definite thrombus, with the greatest dimensions ≤ 1/2 the vessel diameter; in grade 3, there is a definite thrombus but with greatest linear dimension > 1/2 but < 2 vessel diameters; and in grade 4, there is definite thrombus, with the largest dimension ≥ 2 vessel diameters^16^. If the vessel is occluded, the thrombus burden was graded into one of the previous grades after flow achievement either with wire crossing or a small balloon (1.5 mm diameter) passage or dilatation. Intracoronary thrombus burden grade 4 was considered a high thrombus burden.

The final flow in the infarcted related artery after PPCI was visually calculated according to the TIMI study classification^17^. The flow of TIMI was graded as follows: TIMI flow Grade 0: no antegrade flow; TIMI flow Grade I: Partial contrast penetration beyond the occluded segment with incomplete distal filling; TIMI flow Grade II: Patent epicardial artery with delayed contrast filling of the entire distal artery; TIMI flow Grade III: patent epicardial artery with normal flow. TIMI flow grade < III was defined as slow/no-reflow.

The patients were analyzed to diagnose FH according to the DLCN criteria^2^.All studied patients were examined by transthoracic two-dimensional echocardiography using a GE VIVDE S5 ultrasound system device during their hospital stay. Left ventricular ejection fraction (LV EF) was assessed using the biplane method of disks (modified Simpson method) and the left ventricular wall motion score index (WMSI) was calculated^18^. In-hospital major adverse cardiovascular events (MACE), which is defined as a composite of mortality, heart failure, re-infarction, and stroke, were recorded.

## Diagnosis of familial hypercholesterolemia

Familial hypercholesteremia was diagnosed using DLCN criteria (Table 1). DLCN criteria are based on family history of premature CVD in first-degree relatives, their own CVD history, physical examination findings such as tendon xanthomas or corneal arch, among other parameters, and untreated LDL-C levels^2^. In this study, genetic testing was not carried out. DLCN score was calculated and a total point score of > 8 is considered definite FH, 6–8 is probable FH, 3–5 is possible FH, and < 3 is unlikely FH (Table 1).

**Table 1:** The Dutch lipid clinic network criteria and distribution of the studied patients.

Variable	Distribution	Points	Studied patients	Unlikely FH (< 3) 418 (60.6)	Possible FH (3–5) 176 (25.5)	Probable FH (6–8) 90 (13.0)	Definite FH (> 8) 6 (0.9)
1. Family history	First-degree relative with known premature coronary and/or vascular disease (men aged < 55 years, women aged < 60 years) or with known LDL-C above the 95th percentile for age and gender.	1	446 (64.6)	259 (58.1)	112 (25.1)	69 (15.5)	6 (1.3)
	First-degree relative with tendon xanthoma and/or Arcus Cornelis, or children < 18 years with LDL-C above the 95th percentile for age and gender.	2	15 (2.2)	0 (0)	0 (0)	15 (100)	0 (0)
2. Clinical History	Patients with premature CAD (men aged < 55 years, women aged < 60 years)	2	8 (1.2)	1 (12.5)	0 (0)	6 (75.0)	1 (12.5)
	Patient with premature cerebral/peripheral vascular disease (men aged < 55 years, women aged < 60 years)	1	115 (16.7)	50 (43.5)	35 (30.4)	30 (26.1)	0 (0)
3. Physical Examination	Tendon xanthomata	6	0 (0)	0 (0)	0 (0)	0 (0)	0 (0)
	Arcus Cornelis before the age of 45 years	4	3 (0.4)	0 (0)	1 (33.3)	0 (0)	2 (66.7)
4. LDL-C levels	< 155 mg/dl	0	67 (9.7)	66 (98.5)	0 (0)	1 (1.5)	0 (0)
	155–189 mg/dl	1	352 (51.0)	352 (100)	0 (0)	0 (0)	0 (0)
	190–249 mg/dl	3	177 (25.7)	0 (0)	171 (96.6)	5 (2.8)	1 (0.6)
	250–329 mg/dl	5	90 (13.0)	0 (0)	5 (5.6)	84 (93.3)	1 (1.1)
	≥ 330 mg/dl	8	4 (0.6)	0 (0)	0 (0)	0 (0)	4 (100)
DLCN score	0		37 (3.9)	27 (6.5)			
	1		119 (17.2)	119 (28.5)			
	2		272 (39.4)	272 (65.0)			
	3		26 (3.8)		26 (14.8)		
	4		143 (20.7)		143 (81.2)		
	5		7 (1.0)		7 (4.0)		
	6		55 (8.0)			55 (61.1)	
	7		30 (4.3)			30 (33.3)	
	8		5 (0.7)			5 (5.6)	
	9		4 (0.6)				4 (66.7)
	10		2 (0.3)				2 (33.3)

Data are expressed in the form of frequency (%).

CAD: coronary artery disease; DLCN: Dutch lipid clinic network; LDL-C: low-density lipoprotein cholesterol; FH: familial hypercholesterolemia.

### Statistical analysis

Data were verified, coded, and analyzed using SPSS (Statistical Package for the Social Sciences, version 24, IBM, and Armonk, New York). Descriptive statistics; means, standard deviations, medians, ranges, frequency, and percentages were calculated. Test of significances, Chi-square/Fisher’s Exact/Monte Carlo Exact test was used to compare the difference in the distribution of frequencies among different groups. For continuous variables, an independent sample t-test test was carried out to compare the means between groups. The correlation was performed using the Spearman Rank correlation coefficient. Univariate logistic regression analysis was calculated to investigate the independent significant predictors of high thrombus burden (Odds Ratio (OR), 95% confidence interval (CI), and p-value). Predictors with proven statistical significance from the univariate analyses were further included in the multivariable logistic regression models to investigate the adjusted significant predictors of high thrombus burden (adjusted OR, 95% CI, and p-value). A p-value less than 0.05 was considered significant.

## Results

**Fig. 2:**
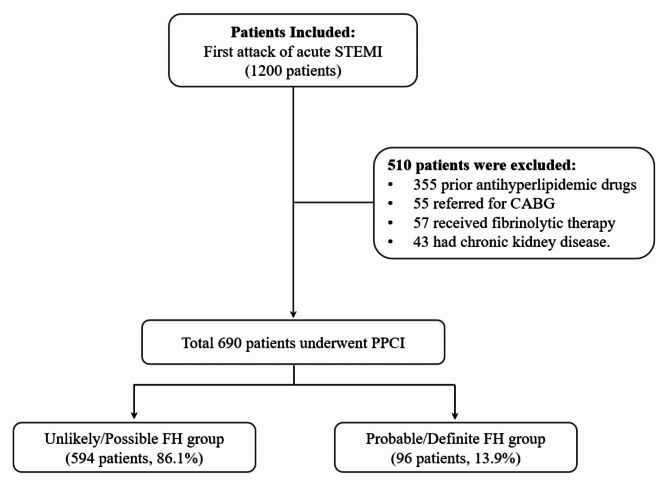
Flow chart of the study population.

During the period of the study, 1,200 patients were presented with the first attack of acute STEMI (type 1 myocardial infarction) and assessed for eligibility. A total of 690 patients were in the final analysis of the present study (Fig. 2). The majority of studied patients were male (501 patients, 72.6%) with an overall mean age of 54 ± 12 years. Nearly, a quarter of them had hypertension, and diabetes mellitus (22.3%, and 25.2%, respectively), and 26.4% were smokers. Table 2 shows the rest of the total patients’ characteristics.

**Table 2:** Patients’ characteristics and in-hospital MACE of total studied patients, unlikely/possible FH, and probable/definite FH groups.

Variables	Total (*n* = 690)	Unlikely/possible FH (*n* = 594)	Probable/definite FH (*n* = 96)	*P*-value
Age (years)	56.42 ± 11.72	58.02 ± 10.17	46.75 ± 15.43	< 0.001
Male sex (%)	501 (72.6)	427 (71.9)	74 (77.1)	0.289
Male < 55 years (%)	213 (30.9)	159 (26.8)	54 (56.2)	< 0.001
Female < 60 years (%)	66 (9.6)	53 (8.9)	13 (13.5)	0.153
Smoking (%)	181 (26.2)	160 (26.9)	21 (21.9)	0.296
DM (%)	174 (25.2)	153 (25.8)	21 (21.9)	0.416
Hypertension (%)	154 (22.3)	140 (23.6)	14 (14.6)	0.050
History of premature TIA/stroke/PAD (%)	115 (16.7)	85 (14.3)	30 (31.2)	< 0.001
Family history of premature CAD and/or vascular disease (%)	446 (64.6)	371 (62.5)	75 (78.1)	0.003
ECG location of STEMI on presentation				
Anterior STEMI (%)	412 (59.7)	350 (58.9)	62 (64.6)	0.181
Inferior STEMI (%)	259 (37.5)	228 (38.4)	31 (32.3)	
Infero-posterior STEMI (%)	9 (1.3)	9 (1.5)	0 (0.0)	
Lateral STEMI (%)	10 (1.4)	7 (1.2)	3 (3.1)	
Killip class on presentation				
Class I (%)	624 (90.4)	536 (90.2)	88 (91.7)	0.776
Class II (%)	36 (5.2)	32 (5.4)	4 (4.2)	
Class III (%)	14 (2.0)	13 (2.2)	1 (1.0)	
Class IV (%)	16 (2.3)	13 (2.2)	3 (3.1)	
LDL-C (mg/dl)	193.3 ± 44.7	179.5 ± 28.2	278.5 ± 32.5	< 0.001
Echocardiographic measures				
LV EF	49.1 ± 8.8	49.2 ± 8.9	48.7 ± 7.8	0.592
LV WMSI	1.39 ± 0.2	1.39 ± 0.2	1.40 ± 0.2	0.743
Length of hospital stay (days)	2.61 ±0.7	2.62 ± 0.7	2.51 ± 0.7	0.123
In-hospital MACE (%)	70 (10.1)	57 (9.6)	13 (13.5)	0.235
Heart Failure (%)	50 (7.2)	41 (6.9)	9 (9.4)	0.386
Mortality (%)	17 (2.5)	14 (2.4)	3 (3.1)	0.652
Re-infarction (%)	4 (0.6)	3 (0.5)	1 (1.04)	0.452
Stroke (%)	5 (0.7)	4 (0.7)	1 (1.04)	0.528

Data are expressed in the form of mean ± standard deviation or frequency (%).

CAD: coronary artery disease; DM: diabetes mellitus; FH: familial hyperlipidemia; LDL-C: low-density lipoprotein cholesterol; LV EF: left ventricular ejection fraction; MACE: major adverse cardiovascular events; PAD: peripheral arterial disease; STEMI: ST elevation myocardial infarction; TIA: transient ischemic attack; LV WMSI: left ventricular wall motion score index.

Based on DLCN criteria for diagnosis of FH, it was unlikely in 418 (60.6%) patients, possible in 176 (25.5%) patients, probable in 90 (13.4%) patients, and 6 (0.9%) patients reached a definite diagnosis (Table 1). Therefore, the study population was classified into two groups: unlikely/possible FH group (594 patients, 86.1%) and probable/definite FH group (96 patients, 13.9%). Probable/definite FH patients were significantly younger, had a higher incidence of males < 55 years, lower incidence of hypertension, and, as expected, had a prominent family history of premature CAD and/or vascular disease compared with unlikely/possible FH patients (*p* < 0.001, < 0.001, 0.05, and < 0.001 respectively) (Table 2). On admission, the location of STEMI was comparable for both groups (*p* = 0.392), and the majority of patients in both groups presented with anterior STEMI. Moreover, the Killip clinical class of patients matched in both groups (*p* = 0.776) with more than 90% being class I in each group. In addition, LV EF was mildly reduced in both groups to the same extent with similar LV WMSI. The length of hospital stay was similar for both groups with a median length of stay of 3 (2–7) days. In-hospital MACE was similar in both groups (9.6% vs. 13.5%, *p* = 0.235).

**Fig. 3:**
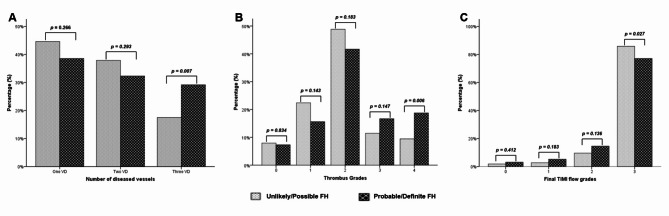
Bar chart of the number of diseased vessels (**A**), thrombus grades (**B**), and final TIMI flow grades (**C**) distribution in unlikely/possible FH and probable/definite FH groups.

**Fig. 4:**
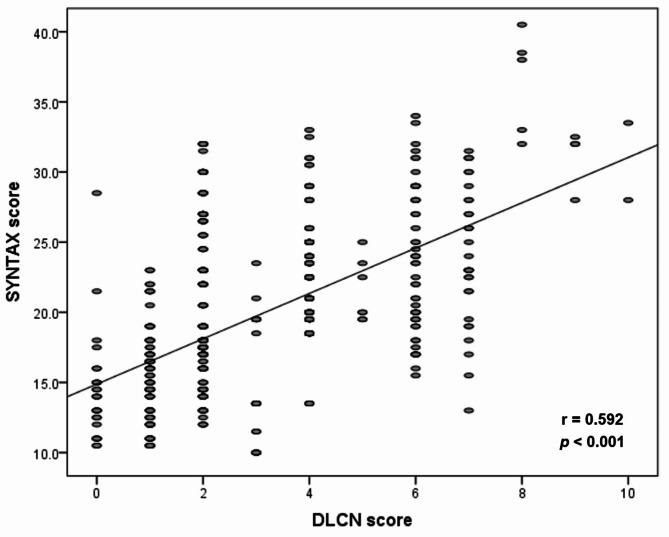
Correlation between Syntax Score and DLCN score among the total studied patients.

Patients’ angiographic and procedure characteristics are shown in Table 3. No significant difference between the unlikely/possible FH and the probable/definite FH groups was found for the location of the infarcted-related artery (*p* = 0.462). Compared to the unlikely/possible FH group, patients in the probable/definite FH group had a higher Syntax score (24.91 ± 5.8 vs. 18.66 ± 4.0, *p* < 0.001) and more frequent three-vessel disease (29.2% vs. 17.5%, *p* = 0.007) (Fig. 3A). Moreover, Fig. 4 shows that the DLCN score had a significant positive moderate correlation with the Syntax score (*r* = 0.592, *p* < 0.001) among the total studied patients. In addition, a significantly high thrombus burden and final TIMI slow/no-reflow were found among patients of the probable/definite FH group compared with the unlikely/possible FH group (*p* = 0.006 for the former and *p* = 0.027 for the latter) (Fig. 3B & C, respectively) with a trend toward the use of Glycoprotein IIb/IIIa inhibitors in patients of the probable/definite FH group (*p* = 0.07). Moreover, in multivariate analysis of the studied patients, males < 55 years, LDL-C level, probable/definite FH, and the number of diseased vessels were independent predictors of high thrombus burden (p-value 0.02, < 0.001, 0.004 and 0.019 respectively) (Table 4).

**Table 3:** Patients’ angiographic and procedure characteristics of total studied patients, unlikely/possible FH, and probable/definite FH groups.

Variables	Total (*n* = 690)	Unlikely/possible FH (*n* = 594)	Probable/definite FH (*n* = 96)	*P*-value
Infarcted-related artery				
LAD	413 (59.9)	349 (58.8)	64 (66.7)	0.462
LCx	98 (14.2)	89 (15.0)	9 (9.4)	
RCA	167 (24.2)	144 (24.2)	23 (24.0)	
Ramus	9 (1.3)	9 (1.5)	0 (0.0)	
LM	2 (0.3)	2 (0.3)	0 (0.0)	
Not identified	1 (0.1)	1 (0.2)	0 (0.0)	
Number of diseased vessels				
One vessel disease	302 (43.8%)	265 (44.6%)	37 (38.5%)	0.026
Two vessel disease	256 (37.1%)	225 (37.9%)	31 (32.3%)	
Three vessel disease	132 (19.1%)	104 (17.5%)	28 (29.2%)	
Syntax score	19.53 ± 5.5	18.66 ± 4.9	24.91 ± 5.8	< 0.001
Thrombus burden grade				
0	54 (7.8%)	47 (7.9%)	7 (7.3%)	0.024
1	174 (21.3%)	133 (22.4%)	15 (15.6%)	
2	331 (48%)	290 (48.8%)	40 (41.7%)	
3	84 (12.2%)	68 (11.4%)	16 (16.7%)	
4	74 (10.7%)	56 (9.4%)	18 (18.8%)	
High thrombus burden	74 (10.7%)	56 (9.4%)	18 (18.8%)	0.006
Thrombus aspiration device	9 (1.3%)	5 (0.8%)	4 (4.2%)	0.008
Number of used stents	1.16 ± 0.5	1.17 ± 0.5	1.11 ± 0.6	0.354
Final TIMI flow grade				
0	14 (2)	11 (1.9)	3 (3.1)	0.035
I	21 (3)	16 (2.7)	5 (5.2)	
II	71 (10.3)	57 (9.6)	14 (14.6)	
III	584 (84.6)	510 (85.9)	74 (77.1)	
Final TIMI flow slow/no-reflow	106 (15.4%)	84 (14.1%)	22 (22.9%)	0.027
Glycoprotein IIb/IIIa inhibitors	259 (37.5%)	215 (36.2%)	44 (45.8%)	0.070

Data are expressed in the form of mean ± standard deviation or frequency (%).

LAD: left anterior descending; LCx: left circumflex; LM: left main; RCA: right coronary artery; TIMI: thrombolysis in myocardial infarction.

**Table 4:** Univariate and multivariate logistic regression analysis of predictors for high thrombus burden among the total studied patients.

Variables	Univariate analysis		Multivariable analysis	
	OR (95% CI)	*p*-value	Adjusted OR (95% CI)	*p*-value
Premature CAD (male < 55 - female < 60)	1.642 (1.013–2.664)	0.044		
Male < 55	1.950 (1.194–3.183)	0.008	3.490 (1.182–10.303)	0.024
ECG location of STEMI on presentation				
Anterior STEMI	0.392 (0.239–0.643)	< 0.001		
Inferior STEMI	1.887 (1.162–3.063)	0.010		
Posterior STEMI	8.855 (2.501–31.353)	0.001		
LDL-C (mg/dl)	1.015 (1.010–1.019)	< 0.001	1.022 (1.012–1.033)	< 0.001
Probable/definite FH	2.217 (1.239–3.967)	0.007	5.675 (1.760–18.297)	0.004
Infarcted-related artery				
LAD	0.389 (0.237–0.638)	< 0.001		
LCx	2.154 (1.205–3.849)	0.010		
RCA	1.825 (1.093–3.048)	0.022		
Number of diseased vessels	2.801 (2.005–3.912)	< 0.001	1.810 (1.102–2.971)	0.019
Syntax score	1.129 (1.083–1.177)	< 0.001		

CAD: coronary artery disease; CI: confidence interval; DLCN: Dutch Lipid Clinic Network; ECG: electrocardiogram; LAD: left anterior descending; LCx: left circumflex; LDL-C: low-density lipoprotein cholesterol; OR: odds ratio; RCA: right coronary artery; STEMI: ST-elevation myocardial infarction.

## Discussion

In the present study, the characteristics of patients with probable/definite FH hospitalized for STEMI differ from patients with unlikely/possible FH; they were younger (12 years younger) with predominantly male patients with premature CAD (< 55 years), lower incidence of hypertension, and had a higher prevalence of history and/or family history of premature CVD. FH is an autosomal dominant inherited genetic disorder that has severely elevated LDL-C levels since birth^19^. This elevated LDL-C level leads to accelerated atherosclerosis that consequently raises the risk of premature CAD^20^. Thence in our study, STEMI patients with probable/definite FH were younger age and had a higher incidence of a history of premature CVD. FH is inherited in families in an autosomal dominant^19^, therefore it was expected that a family history of premature CVD was significantly prevalent in probable/definite FH. These findings are supported by the results of many previous studies and registries^11,21–23^. In general, there is no sex difference in patients with FH^7,23^. However, we found that premature CAD was significantly more prevalent among males (age < 55 years) with probable/definite FH. These findings are comparable to those previously published data from the CASCADE-FH registry, British Columbia FH Registry, and other authors^24–26^. British Columbia FH Registry with a diagnosis of probable or definite FH according to DLCN Criteria, the age of the first cardiovascular event in males was significantly younger than in females (*p* = 0.01)^25^. Hypertension prevalence increased progressively with age. Based on data from the Egyptian Ministry of Health and Population: the age-standardized prevalence of hypertension for individuals aged 35 to 49 years and 50 to 59 years were around 12.1% and 27.2%, respectively^27,28^. In the present study, the mean age in unlikely/possible and probable/definite FH groups was 58 and 46 years, respectively. This goes along with our finding that hypertension was significantly prevalent among unlikely/possible FH patients (23.6% and 14.6%, *p* = 0.5) which have already been reported in several cohorts^7,11,12,21,29^.

Our study revealed that the clinical presentation and in-hospital course of STEMI patients who underwent PPCI were similar in both unlikely/possible FH and probable/definite FH groups. In both groups, most of the patients presented with anterior STEMI, and the majority had functional Killip class I with mildly reduced EF. Moreover, in-hospital MACE did not differ in both groups. In the general population, anterior wall STEMI is the common location and this is not different in patients with FH as our study demonstrated. RICO study, which is a survey that has included all consecutive patients > 18 years hospitalized for myocardial infarction in the region of Côte d’Or (France) since 2001, supports our findings as showed that anterior wall STEMI was the most common site in patients with no FH and FH (65% for the former and 56% for the latter, *p* = 0.154)^12^. Moreover, similar to our study, the French registry of Acute ST-elevation and non-ST-elevation Myocardial Infarction (FAST-MI 2005 and 2010) revealed that the majority of the patients with no FH and FH presented with functional Killip class I (84% versus 87.5% respectively, *p* = 0.246 )^21^. Our study showed that patients’ EF was mildly reduced in both groups (49 versus 48, *p* = 0.592). Other studies such as the RICO study and FAST-MI 2005 and 2010 registry^12,21^ showed good EF (55 versus 55 for the former and 52 versus 53 for the latter) in both groups because these studies included patients with STEMI and non-STEMI and our study included only STEMI patients. In the present study, patients with probable/definite FH had a similar risk for in-hospital MACE as those with unlikely/possible FH which may be attributed to quite comparable patients’ comorbidities in both groups. Few studies addressed the in-hospital complications or MACE as a part of the long-term outcome of patients with acute coronary syndrome and FH^7,12,21,29,30^. Some of these studies^12,21,29^ demonstrated similar in-hospital complications (heart failure, recurrent myocardial infarction, stroke, and death) as the studied population baseline characteristics in both non-FH and FH groups were comparable. The other studies^7,30^ showed few differences regarding in-hospital outcomes compared to our study as they showed a higher in-hospital all-cause mortality in patients with non-FH. They included a heterogeneous population (STEMI, non-STEMI, and unstable angina) and the patients with non-FH were older and exposed to many comorbidities compared to those with FH.

Few studies tackled the angiographic characteristics of patients with FH ^9–12,31−34^. The present study boosts the findings of the previous studies and highlights another aspect of the angiographic features of patients with FH and presented with STEMI. In the present study, the severity of CAD is characterized by frequent three-vessel disease (Fig. 3A), and the anatomical complexity of CAD is distinguished by a high Syntax score (Table 3) in patients with probable/definite FH. These findings suggest that the elevated cholesterol burden in FH, which starts at an early age, and high DLCN score are associated with CAD severity and anatomical complexity. This speculation is supported by the significant positive correlation between the DLCN score and the Syntax score in the present study (Fig. 4). These data are matched with previous studies’ findings in which the severity of CAD was assessed either by frequency of multi-vessel CAD, Gensini score^31^, or both. They reported frequent multi-vessel CAD in FH patients, while non-FH patients had more frequent one-vessel CAD^12,32–35^. Two other studies showed that the Gensini score was significantly increased with the definite/probable FH diagnosis in both men and women compared to possible and unlikely FH^9,10^. In addition, Wang et al. stated that FH was associated with a significantly higher prevalence of multi-vessel CAD and a higher Gensini score^11^. Only three studies address the anatomical complexity of CAD in patients with FH^11,12,35^. Wang et al.^11^ assessed only chronic total occlusion and reported its higher prevalence among FH patients compared with non-FH patients (45.9% vs. 29.9%). Yasuda and his colleagues^35^ defined complex lesions by the presence of one or more of the following features: irregular borders, abrupt lesion edges perpendicular to the vessel wall, ulceration, or the presence of a thrombus. They found that patients with FH had more prevalent total obstructive and complex lesions compared with non-FH patients (56% vs. 11%; *p* < 0.01 for the former and 38% vs. 15%; *p* < 0.01 for the latter). Yao and Coworkers^12^ focused on the complexity of the lesions using Syntax score and prespecified lesions complex criteria from the CHAMPION-PHOENIX and DAPT studies^36,37^. They showed that patients in the FH group had a higher initial Syntax score (*p* = 0.005), more multiple lesions (*p* = 0.022), bifurcation lesions (*p* = 0.017), and calcified lesions (*p* = 0.033) with a non-significant trend towards more multiple complex lesions > 1 (*p* = 0.053) compared to the non-FH group. These results are in line with our findings as we believe that the Syntax score included all components of the lesion’s complex criteria in the CHAMPION-PHOENIX and DAPT studies. Therefore, the Syntax score expresses the anatomical complexity of CAD. These studies were retrospective, included patients with chronic coronary syndrome, acute coronary syndrome (STEMI, non-STEMI, and unstable angina), or acute myocardial infarction (STEMI, and non-STEMI), and included patients on statin therapy that affect the lipogram compared to our study that was a prospective study, focused only on patients with STEMI, and excluding patients on statin therapy. In addition, some DLCN criteria such as tendon xanthomas, corneal arches, and/or family history of premature CAD and/or vascular disease were not collected in these studies.

To the best of our knowledge, the current study is the first to evaluate the thrombus burden and its predictors in patients with probable/definite FH hospitalized for STEMI who underwent PPCI. In the current study, the high thrombus burden was significantly more frequent in patients with probable/definite FH, and LDL-C level was one of the predictors of high thrombus burden. Elevated LDL-C promotes lipid-rich core atheromatous plaque with a honeycomb-like accumulation of foam cells, cholesterin clefts, and blood infiltration from the lumen to atheromatous plaque increasing intra-plaque pressure and causing atheromatous plaque rupture with intracoronary thrombosis development^38^. This is the basic pathophysiologic mechanism in patients with acute coronary syndrome. There are several speculated potential reasons for high thrombus burden in patients with FH and STEMI, primarily related to elevated LDL-C and its effects on the vascular system. Human LDL-C can be chromatographically divided into 5 subfractions (L1-L5) with increasing electronegativity^39^. The most electronegative L5 is significantly higher in FH compared with normal subjects (2–5% vs. 0.6% of total plasma LDL-C, respectively)^40^. L5 in STEMI patients increased adenosine 5 diphosphate, platelet P-selectin expression, and GP IIb/IIIa activation, thereby triggering platelet activation and aggregation with thrombus formation^41^. Moreover, LDL-C consists of aggregating LDL-C, and Lp(a) constitutes 30–45% of total LDL-C^42^. Patients with FH, especially those with CVD, had higher Lp(a) plasma levels^43^. In addition to the pro-atherosclerotic effects of Lp(a), it has anti-fibrinolytic and pro-thrombotic properties through impairment of plasmin generation and stimulation of platelet aggregation^44^. Therefore, elevated Lp(a) is associated with an increased risk of high thrombus burden in STEMI patients^45^. Furthermore, it has been shown that the platelet aggregation parameters and mean platelet volume in patients with FH were significantly increased compared to patients with non-FH^46,47^. Hence, these findings suggest that increased platelet reactivity may provoke thrombus formation and increase thrombus burden in patients with FH. In addition, plasma fibrinogen and coagulation factor VIII levels are significantly higher in patients with FH and CAD^48^, and polymorphism of the LDL-receptor, the main mechanism of high LDL-C in FH families, is related to increased coagulation factor VIII and accelerated coagulation^49^.

### Limitations

The current study has some limitations that one has to consider. Firstly, this study was a single-center study so a multicenter large-scale study may be more informative with different groups. Secondly, this study was observational, as with any observational analysis, we were unable to conclude causal relationships correlated to measured and unmeasured confounding biases. Nevertheless, a large sample size was used to increase the power of the study to identify real effects. Thirdly, genetic analysis was not performed to confirm FH in this study, which may have contributed to some degree of underdiagnosis of FH. However, some studies showed that definite FH diagnosis based on DLCN criteria offers a comparable detection rate to genetic analysis^50,51^.

In conclusion, STEMI patients with FH were more prone to be younger males, having a history of CAD and a family history of premature CVD with a lower incidence of hypertension. In addition, they were associated with severe CAD with frequent multivessel CAD and high anatomical complexity of CAD. Furthermore, high thrombus burden was frequently encountered among patients with FH with LDL-C level as one of its predictors besides males < 55 years, and the number of diseased vessels.

## Data Availability

The datasets used and/or analyzed during the current study are available from the corresponding author upon reasonable request.

## Abbreviations

CAD: coronary artery disease
CI: confidence interval
CVD: cardiovascular disease
DLCN: Dutch Lipid Clinic Network
ECG: electrocardiogram
FH: familial hypercholesterolemia
LDL-C: low-density lipoprotein cholesterol
LV EF: left ventricular ejection fraction
MACE: major adverse cardiovascular events
OR: odds ratio
PPCI: primary percutaneous coronary interventions
STEMI: ST-elevation myocardial infarction
TIMI: Thrombolysis in Myocardial Infarction
WMSI: wall motion score index
